# The Tumor Microbiome as a Predictor of Outcomes in Patients with Metastatic Melanoma Treated with Immune Checkpoint Inhibitors

**DOI:** 10.1158/2767-9764.CRC-23-0170

**Published:** 2024-08-08

**Authors:** Caroline E. Dravillas, Samuel S. Coleman, Rebecca Hoyd, Griffin Caryotakis, Louis Denko, Carlos H.F. Chan, Michelle L. Churchman, Nicholas Denko, Rebecca D. Dodd, Islam Eljilany, Sheetal Hardikar, Marium Husain, Alexandra P. Ikeguchi, Ning Jin, Qin Ma, Martin D. McCarter, Afaf E.G. Osman, Lary A. Robinson, Eric A. Singer, Gabriel Tinoco, Cornelia M. Ulrich, Yousef Zakharia, Daniel Spakowicz, Ahmad A. Tarhini, Aik Choon Tan, for the exORIEN Consortium

**Affiliations:** 1 Division of Medical Oncology, The Ohio State University Comprehensive Cancer Center, Columbus, Ohio.; 2 Department of Oncological Science, Huntsman Cancer Institute, University of Utah, Salt Lake City, Utah.; 3 Department of Biomedical Informatics, Huntsman Cancer Institute, University of Utah, Salt Lake City, Utah.; 4 Pelotonia Institute for Immuno-Oncology, The Ohio State University Comprehensive Cancer Center, Columbus, Ohio.; 5 Holden Comprehensive Cancer Center, University of Iowa, Iowa City, Iowa.; 6 Aster Insights, Hudson, Florida.; 7 Department of Radiation Oncology, The Ohio State University Comprehensive Cancer Center, Columbus, Ohio.; 8 Department of Internal Medicine, University of Iowa, Iowa City, Iowa.; 9 Department of Cutaneous Oncology, H. Lee Moffitt Cancer Center and Research Institute, Tampa, Florida.; 10 Department of Population Health Sciences, Huntsman Cancer Institute, University of Utah, Salt Lake City, Utah.; 11 Department of Hematology/Oncology, Stephenson Cancer Center of University of Oklahoma, Oklahoma City, Oklahoma.; 12 Department of Biomedical Informatics, The Ohio State University, Columbus, Ohio.; 13 Department of Surgery, University of Colorado School of Medicine, Aurora, Colorado.; 14 Division of Hematology and Hematologic Malignancies, Department of Internal Medicine, University of Utah, Salt Lake City, Utah.; 15 Department of Thoracic Oncology, H. Lee Moffitt Cancer Center and Research Institute, Tampa, Florida.; 16 Division of Urologic Oncology, The Ohio State University Comprehensive Cancer Center, Columbus, Ohio.; 17 Division of Oncology, Hematology and Blood and Marrow Transplantation, University of Iowa, Holden Comprehensive Cancer Center, Iowa City, Iowa.; 18 Department of Immunology, H. Lee Moffitt Cancer Center and Research Institute, Tampa, Florida.

## Abstract

Emerging evidence supports the important role of the tumor microbiome in oncogenesis, cancer immune phenotype, cancer progression, and treatment outcomes in many malignancies. In this study, we investigated the metastatic melanoma tumor microbiome and its potential roles in association with clinical outcomes, such as survival, in patients with metastatic disease treated with immune checkpoint inhibitors (ICI). Baseline tumor samples were collected from 71 patients with metastatic melanoma before treatment with ICIs. Bulk RNA sequencing (RNA-seq) was conducted on the formalin-fixed, paraffin-embedded and fresh frozen tumor samples. Durable clinical benefit (primary clinical endpoint) following ICIs was defined as overall survival ≥24 months and no change to the primary drug regimen (responders). We processed RNA-seq reads to carefully identify exogenous sequences using the {exotic} tool. The age of the 71 patients with metastatic melanoma ranged from 24 to 83 years, 59% were male, and 55% survived >24 months following the initiation of ICI treatment. Exogenous taxa were identified in the tumor RNA-seq, including bacteria, fungi, and viruses. We found differences in gene expression and microbe abundances in immunotherapy-responsive versus nonresponsive tumors. Responders showed significant enrichment of bacteriophages in the phylum *Uroviricota,* and nonresponders showed enrichment of several bacteria, including *Campylobacter jejuni*. These microbes correlated with immune-related gene expression signatures. Finally, we found that models for predicting prolonged survival with immunotherapy using both microbe abundances and gene expression outperformed models using either dataset alone. Our findings warrant further investigation and potentially support therapeutic strategies to modify the tumor microbiome in order to improve treatment outcomes with ICIs.

**Significance:**

We analyzed the tumor microbiome and interactions with genes and pathways in metastatic melanoma treated with immunotherapy and identified several microbes associated with immunotherapy response and immune-related gene expression signatures. Machine learning models that combined microbe abundances and gene expression outperformed models using either dataset alone in predicting immunotherapy responses.

## Introduction

Advances in immunotherapy, including immune checkpoint inhibitors (ICI), have transformed the standard of care for many types of cancer, including melanoma. Although ICIs have improved outcomes for patients with melanoma, many patients suffer from primary or secondary tumor resistance. For example, at 6.5 years, the overall survival (OS) rates with ipilimumab plus nivolumab, nivolumab, and ipilimumab were 49%, 42%, and 23%, respectively, as reported in the pivotal CheckMate 067 trial (1). Furthermore, mechanisms of resistance to immunotherapy remain poorly understood, and many treatments are associated with immune-mediated toxicities. Therefore, there is an urgent need to develop and improve biomarkers predictive of benefit from ICI therapy.

Numerous biomarkers that predict the response of melanoma to ICIs are under investigation, including those based on clinical characteristics, genomics, transcriptomics, and epigenomics. For genomic data, these predictive biomarkers include tumor mutational burden (TMB; ref. 2), neoantigen load (3), genotypes of HLA-I (3, 4), T-cell repertoire (5), aneuploidy (also known as somatic copy number alterations; ref. 6), and germline variations (7). On the other hand, predictive biomarkers derived from transcriptomic data include tumor oncogene expression signatures, such as genes related to MYC (8), WNT/β-catenin (9, 10), or RAS (11) signaling; gene expression profiles within the tumor immune microenvironment (TIME), such as IFNγ-responsive genes (12), chemokines (13, 14), MHC classes I and II (15); and cytotoxic T-cell and effector T-cell (16, 17) gene expression markers that have been reported to be predictive of ICI response in metastatic melanoma. Unfortunately, the predictive power remains low. For example, in terms of prediction of ICI response, TMB, IFNγ-responsive gene signatures, or the combination of TMB and IFNγ gene signatures produce an area under the receiver operating characteristic (AUROC) curve of 0.60 to 0.84 in melanoma cohorts (18).

Recently, high-throughput transcriptome-, genome-, or amplicon-based sequencing data demonstrated an abundance and variety of microbes’ nucleic acids inside tumors (8). In some cases, 100 of negative controls and paraffin-only blocks were sequenced to ensure a thorough understanding of the background signal and reagent contamination. Furthermore, the presence of microbes has been validated using FISH and IHC (19). The microbes showed cancer specificity (9, 12, 13), and blood-based measurements could predict early-stage disease. These findings suggest that microbes observed in high-throughput sequencing data may also correlate with treatment outcomes. Recent efforts to use these microbes as biomarkers showed that although generally less predictive of prognosis than gene expression, when combined with gene expression, they increase the predictive power (20). Furthermore, the tumor microbiome was predictive of chemotherapy response.

**Figure 1:**
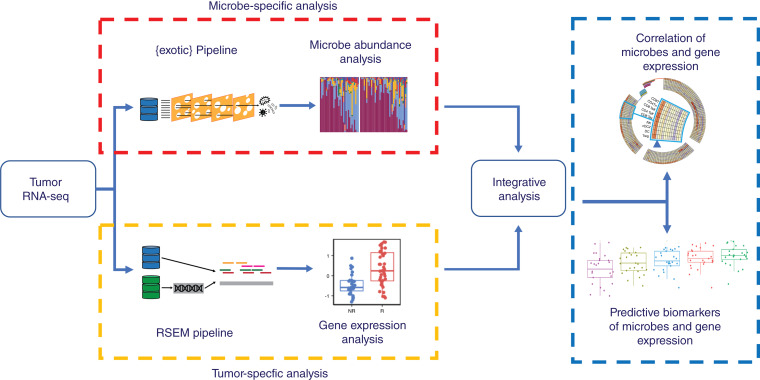
Graphical summary. RNA-seq data from tumor specimens are processed to microbe abundances and human gene expression. Each is associated with ICI response, and then integrative analyses combine them into a model to predict outcomes.

Here, we describe the use of tumor RNA sequencing (RNA-seq) to predict response to ICIs in patients with melanoma (Fig. 1). We demonstrate the presence of microbes within tumors and show different microbial communities in patients whose tumors responded to treatment. We predict treatment response using human gene expression patterns that perform similarly to other ICI response prediction efforts. Finally, we show how the presence of microbes correlates with these signatures, suggesting an interaction with the immune system, and how including tumor microbes in these models improves their predictive accuracy.

## Materials and Methods

### Study design

Established in 2014, the Oncology Research Information Exchange Network (ORIEN) is an alliance of 18 US cancer centers. All ORIEN alliance members utilize a standard institutional review board (IRB)-–approved protocol: Total Cancer Care (TCC). As part of the TCC protocol, participants provide written informed consent to have their clinical data followed over time, undergo germline and tumor sequencing, and be contacted in the future by their provider if an appropriate clinical trial or other study becomes available (21). TCC is a prospective cohort study in which a subset of patients elect to be enrolled in the ORIEN Avatar program, which provides research use only–grade whole-exome tumor sequencing, RNA-seq, germline sequencing, and collection of deep longitudinal clinical data with lifetime follow-up. Nationally, more than 325,000 participants have enrolled in TCC. M2GEN, the commercial and operational partner of ORIEN, harmonizes all abstracted clinical data elements and molecular sequencing files into a standardized, structured format to enable the aggregation of de-identified data for sharing across the network. Data access was approved by the IRB in an Honest Broker protocol (2015H0185) and TCC protocol (2013H0199) in coordination with M2GEN and participating ORIEN members. In this study, we assembled RNA-seq data from the tumor samples of 71 patients with metastatic melanoma treated with ICIs.

### Primary clinical endpoint

In this real-world dataset, no tumor size measurements were available to assess treatment response according to immune response evaluation criteria in solid tumors (iRECIST) criteria or other standardized scores. Instead, in concordance with other studies with similar cohorts (22–24), we defined durable clinical benefit as OS ≥24 months after the start of ICI treatment (hereafter referred to as responders). Therefore, nonresponders survived for <24 months after the initiation of ICI treatment.

### Sequencing methods

ORIEN Avatar specimens underwent nucleic acid extraction and sequencing at HudsonAlpha (Huntsville, AL) or Fulgent Genetics (Temple City, CA). For frozen and optimal cutting temperature (OCT) tissue DNA extraction, Qiagen QIASymphony DNA purification was performed, generating a 213 bp average insert size. For frozen and OCT tissue RNA extraction, the Qiagen RNeasy Plus Mini Kit was used, generating a 216 bp average insert size. For formalin-fixed, paraffin-embedded tissue, a Covaris Ultrasonication FFPE DNA/RNA kit was utilized to extract DNA and RNA, generating a 165 bp average insert size. RNA-seq was performed using the Illumina TruSeq RNA Exome with single library hybridization, cDNA synthesis, library preparation, and sequencing (100 bp paired reads at HudsonAlpha; 150 bp paired reads at Fulgent Genetics) to a coverage of 100 M total reads/50 M paired reads.

### Data processing and gene expression analyses

RNA-seq tumor pipeline analysis was processed according to the workflow outlined below using GRCh38/hg38 human genome reference sequencing and GenCode build version 32. Adapter sequences were trimmed from the raw tumor sequencing FASTQ file. Adapter trimming via k-mer matching was performed along with quality trimming and filtering, contaminant filtering, sequence masking, guanosine-cytosine (GC) filtering, length filtering, and entropy filtering. The trimmed FASTQ file was used as input to the read alignment process. The tumor adapter–trimmed FASTQ file was aligned to the human genome reference (GRCh38/hg38) and the GenCode genome annotation v32 using the STAR aligner. The STAR aligner generates multiple output files for gene fusion prediction and gene expression analysis. RNA expression values were calculated and reported using estimated mapped reads, fragments per kilobase of transcript per million mapped reads, and transcripts per million (TPM) mapped reads at both the transcript and gene levels based on transcriptome alignment generated using STAR. RSEM pipeline and gene expressions were quantified as TPM. Gene expressions were log_2_(TPM + 1)-transformed, and downstream analyses were performed using GE Matrix. To determine differentially expressed genes (DEG) of responders versus nonresponders, we used the *limma* (v. 3.54.0) and *edgeR* (v. 3.40.0) packages, in which genes that have log_2_ fold change greater or less than 1 and adjusted *P* value ≤0.1 were considered significant DEGs. For gene set enrichment analysis (GSEA) of responders vs. nonresponders, we used the Java version of *gsea* (v. 4.3.2) using the gene set permutation of 1000 using Hallmark gene sets or TIMEx cell types. Gene sets or cell types that have adjusted *P* value <0.1 were considered significant. The normalized enrichment score (NES) and adjusted *P* value were provided in the plot.

### Microbe abundance and diversity

RNA-seq reads were used to calculate microbe abundances using the {exotic} pipeline v1.2, which included a 16S validation cohort and described a series of decontamination and filtering steps in detail (25). Briefly, reads were aligned first to the human reference genome, and then unaligned reads were mapped to a database of bacteria, fungi, archaea, viruses, and eukaryotic parasites. The observed microbes were then proceeded through a series of filtering steps to carefully and conservatively remove contaminants, including segmenting the CHM13 human transcriptome into 100 base pairs, with 50 base pair overlaps, running through the exotic pipeline, and filtering out any microbe falsely identified in this process. We used raw counts to calculate diversity measures and for differential abundance analyses using {DESeq2} (26). The stacked bar plot and correlations used a relative abundance value, generated by dividing raw counts by the number of human reads. Diversity measures were estimated by calculating the Shannon and Simpson indices, as well as Chao1, ACE, and inverse Simpson using the R package {vegan} (27).

### Signature and pathway analyses

Gene signature scores were calculated using the IOSig and tmesig R packages. In brief, for each published gene signature, we collected and harmonized gene names using the NCBI Entrez gene number. To quantify the published gene expression score, we first transformed the gene expressions across samples within a cohort into a *Z*-score. Next, we averaged the standardized *Z*-score across the number of genes in the signature, as previously described (15, 28, 29). This score is used to compare responders and nonresponders of immunotherapies within individual cohorts based on the AUROC, as previously described (28). We performed clustering of gene signatures based on the correlation of AUROC across multiple cohorts. Within a cohort of patients, we stratified the patients into “high” or “low” groups based on the mean of the *Z*-score. A Mann–Whitney U test was performed by comparing the two groups to determine the difference, and a FDR of <0.05 was deemed to be significant. The list of published gene signatures is available in Supplementary Table S1.

For pathway analysis, single-sample GSEA (ssGSEA), via the ssGSEA method in the GSVA R package, was utilized to investigate the enriched gene sets in each sample. GSVA was run using the log_2_(TPM + 1) gene expression values with the Gaussian kernel. The Hallmark gene sets, TIMEx cell types, and the collected previously published gene expression signatures were used as the gene sets. The Hallmark gene sets are a curated list of gene sets that signify well-understood pathways that display reliable gene expression (30). The TIMEx cell types are formed from pan-cancer single-cell RNA-seq signatures and focus on illuminating immune cell infiltration from bulk RNA-seq data (31). A Spearman correlation analysis was conducted using the differentially expressed microbe data and the three ssGSEA results. The gene sets were clustered according to the Euclidean distance with complete linkage, whereas the microbes were ordered from the highest to the lowest effect size.

### Prediction of response to treatment outcomes

To assess the predictive ability of the RNA-seq and microbe data for tumor response to ICIs, random forest classifiers were created using the *randomForest* R package. Models were based on five sets of input data: (i) microbe data, (ii) 31–gene signature *Z*-score, (iii) immune-activated gene signature *Z*-score, (iv) microbe and 31–gene signature *Z*-score combined, and (v) microbe- and immune-activated gene signature *Z*-score combined. Models were constructed with 500 trees and fivefold cross-validation. Additionally, 5 seeds were used for each model, resulting in 25 trained models based on each set of input features. The AUROC curve was used to assess the overall performance of the trained models. This metric assesses the model classification accuracy, in which 1 is a perfect classifier and 0.5 is a random classifier. The overall performance for each input feature–based model was taken as the average of the 25 trained models.

### Independent validation datasets

We downloaded two independent melanoma RNA-seq datasets from public repositories: Gide and colleagues (BioProject: PRJEB23709, *n* = 91; ref. 32) and Riaz and colleagues (BioProject: PRJNA356761, *n* = 105; ref. 33). Both datasets contained patients with melanoma receiving various immunotherapies, similar to the ORIEN Avatar training cohort. Raw RNA-seq files were downloaded and aligned as described in RNA-seq data processing and microbe abundance analyses. We used the random forest models developed in the ORIEN Avatar training set and tested the balanced accuracy of these models. The balanced accuracy is the arithmetic mean of sensitivity and specificity which account for imbalanced data.

### Data availability

The Ohio State University IRB approved data access in an Honest Broker protocol (2015H0185) and TCC protocol (2013H0199) in coordination with Aster Insights. The processed data generated in this study are publicly available in Gene Expression Omnibus through the BioProject PRJNA856973. Analysis scripts to regenerate all figures and tables are available at https://github.com/spakowiczlab/exorien-melio.

## Results

### Patient characteristics

From the ORIEN networks, we included 71 patients with metastatic melanoma in this study (IO_NOVA_Mel). The age of the patients in this cohort ranged from 24 to 83 years; 59% were male; and 55% survived >24 months following the initiation of ICI treatment (Table 1). ICI treatments included anti-CTLA4 (18.8% of nonresponders and 41.0% of responders), anti-PD1/PDL1 (62.5% of nonresponders and 56.4% of responders), and anti-CTLA + anti-PD1/PDL1 (18.8% of nonresponders and 2.6% of responders). The mean OS of responders (49.58 months) and nonresponders (10.82 months) was significantly different (*P* value <0.001).

**Table 1:** Patient demographics stratified by response to ICIs

	Nonresponder (*n* = 32)		Responder (*n* = 39)		*P* value
Age [mean (SD)]	57.48 (15.85)		58.62 (13.93)		0.748
Sex = male (%)	18 (56.2)		24 (61.5)		0.835
ICI [*n* (%)]	Anti-CTLA4	6 (18.8)	Anti-CTLA4	16 (41.0)	0.022
	Anti-PD1/PDL1	20 (62.5)	Anti-PD1/PDL1	22 (56.4)	
	Anti-CTLA4 + anti-PD1/PDL1	6 (18.8)	Anti-CTLA4 + anti-PD1/PDL1	1 (2.6)	
Sample collected within 1 year of ICI start = Yes (%)	24 (75.0)		29 (74.4)		1.000
OS [mean (SD)] months	10.82 (6.23)		49.58 (19.24)		<0.001

### Gene expression analysis and its association with response to ICIs

**Figure 2:**
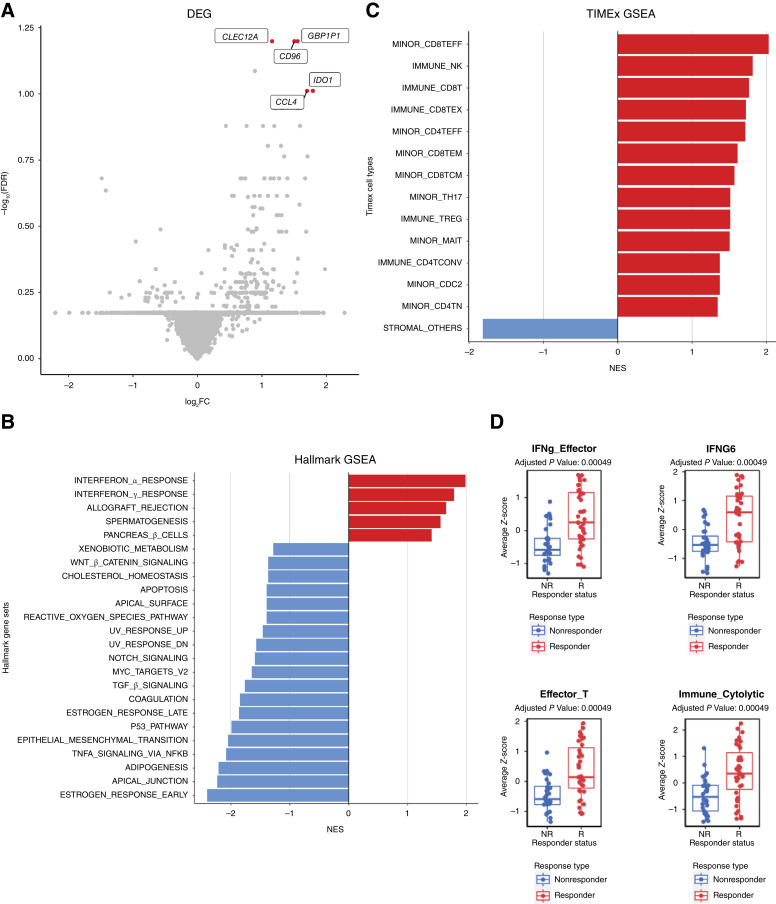
Immune-related gene expression associates with the response to ICIs. **A,** Gene expression differences between the tumors responsive (right) and nonresponsive (left) to ICI treatment. Significantly different genes after FDR correction are colored and labeled. **B** and **C,** GSEA comparing responders vs. the nonresponders using the Hallmark gene set and TIMEx cell types. FDR < 0.1 was used as the cutoff. **D,** Mann–Whitney comparison of responders and nonresponders for signatures reaching the 0.05 FDR threshold.

The gene expression profiles for the 71 patients with metastatic melanoma treated with ICIs were obtained from ORIEN. We performed DEG analysis and identified five genes (*CLEC12A*, *GBP1P1*, *CD96*, *CCL4*, and *IDO1*) that were overexpressed in the responders compared with the nonresponders with log_2_ fold change >1 and adjusted *P* value <0.1 (Fig. 2A; Supplementary Table S2). Interestingly, these five genes were involved in immune modulation and have been previously identified in other studies as predictive biomarkers associated with responders to ICIs. For example, *CCL4* has been previously identified as a biomarker in the 12-chemokine signature (13, 14), as well as other gene signatures predictive of neoadjuvant ipilimumab response (34). *IDO1* has been identified as a key marker in the IFNγ signature (12), as well as other gene signatures predictive of response to ICIs in lung cancer (35). *CD96* is a marker that estimates CD8^+^ T-cell infiltration (36, 37). *CD96* and *TIGIT* along with the costimulatory receptor *CD226* form a pathway that affects the immune response in an analogous way to the CD28/CTLA4 pathway (38). *CLEC12A* (39, 40) and *GBP1P1* (41, 42) were identified in immune-related gene expression signatures predictive of ICI responses.

Next, we investigated which gene sets and pathways were enriched or depleted in responders to ICIs. We performed GSEA using the MSigDB Hallmark gene sets on the RNA-seq and found that several immune-related gene sets were significantly enriched in responders (Fig. 2B), for example, IFNα response (NES = 1.98; FDR < 0.001), IFNγ response (NES = 1.79; FDR < 0.001), and allograft rejection (NES = 1.65; FDR = 0.002). The other two gene sets enriched in responders were spermatogenesis (NES = 1.56; FDR = 0.005) and the pancreas β cell gene sets (NES = 1.40; FDR = 0.036). In contrast, many cell-intrinsic gene sets were enriched in ICI nonresponders, as shown in Fig. 2B. The GSEA results identified in this cohort are similar to previously published studies (28).

We next hypothesized that tumor-infiltrating immune cells could associate with responses to ICIs. To test this hypothesis, we performed cell-type deconvolution of the bulk RNA-seq using CIBERSORT. From CIBERSORT results, we observed that responders had significantly (*P* value <0.05) higher abundances of CD8^+^ T cells, activated CD4^+^ memory T cells, activated NK cells, and M1 macrophages relative to nonresponders, who were shown to have a significantly higher amount of resting mast cells (Supplementary Fig. S1). Similarly, when we performed GSEA using TIMEx gene sets, we observed that 13 CD4^+^-, CD8^+^-, and NK-related cell types were enriched in responders (FDR < 0.1), whereas the stromal cell type was enriched in nonresponders (Fig. 2C). This suggests that the tumor microenvironment (TME) of responders had an “immune-inflamed” phenotype, whereas nonresponders had either “immune-excluded” or “immune-desert” TME phenotypes.

To further delineate the immune phenotypes of responders versus nonresponders of ICIs, we used previously published gene signatures. We collected 30 gene expression signatures from the literature that have been implicated to be predictive of ICIs (28). By performing a *Z*-score for each signature and associating them with responders versus nonresponders, we identified 16 gene signatures (Supplementary Fig. S2) in which high *Z*-scores are associated with ICI responsiveness in this cohort (FDR <0.05), and the top four gene signatures are illustrated in Fig. 2D. These 16 gene signatures were related to immune activation and inflammation signatures (Supplementary Fig. S2; ref. 28).

We next used our recently developed IOSig portal (28) to evaluate the predictive values of these 16 gene signatures in our ORIEN cohort (IO_NOVA_Mel), as well as 22 other melanoma cohorts treated with ICIs. We used AUROC to assess the predictive value of these signatures. For the 16 gene signatures, the AUROC values ranged from 0.78 to 0.66 in the IO_NOVA_Mel cohort (Supplementary Fig. S3; Supplementary Table S3). On average, the AUROC values for these 16 gene signatures ranged from 0.61 to 0.68 in the separate 22 melanoma cohorts (Supplementary Fig. S3; Supplementary Table S3).

### The melanoma tumor microbiome and its association with response to ICIs

**Figure 3:**
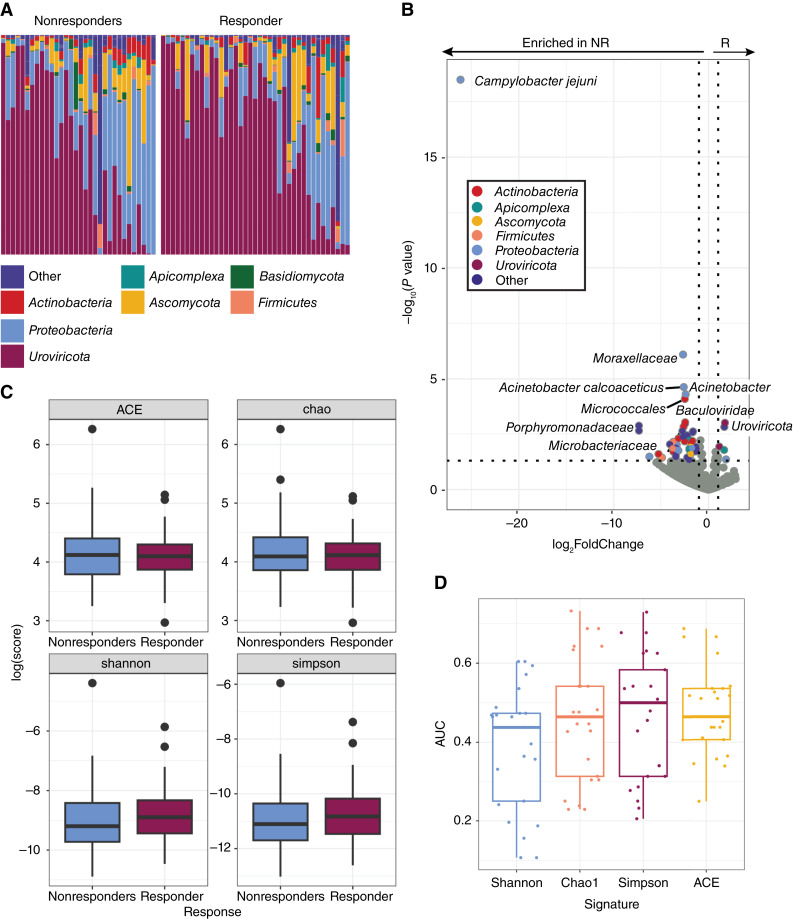
Melanoma tumors that respond to ICIs have a distinct tumor microbiome. **A,** The relative abundances of the tumor microbiome at the phylum level showed wide intersample variation in the abundances of *Proteobacteria* (blue) and viruses [*Uroviricota* (maroon)], without gross differences between nonresponders (NR) and responders (R). **B,** Differential abundance analysis of taxa found within tumor RNA-seq data. Colored points represent significantly (*P* value <0.05) enriched taxa with a high (>1.00) fold difference in abundance between responders and nonresponders to ICIs. **C,** The diversity of the tumor microbiome between responders and nonresponders shows no significant differences. **D,** The diversity of the microbiome is a poor predictor of outcomes.

Exogenous taxa were identified in the tumor RNA-seq, including bacteria, fungi, and viruses. A total of 54 phyla were observed, with *Firmicutes* being the most abundant phylum, followed by *Uroviricota* (Fig. 3A). Within the tumors responsive to immunotherapy, we found a significant enrichment of the *Uroviricota* phylum. Comparatively, the cohort of nonresponsive tumors was found to have significant intratumoral enrichment of bacteria and viruses, including *Campylobacter jejuni*, *Acinetobacter calcoaceticus*, and the *Baculoviridae* family (Fig. 3B; Supplementary Table S4). In order to provide further validation, we conducted an analysis checking The Cancer Genome Atlas data for the microbes most significantly enriched in our response groups. All of the microbes that best discriminate between SKCM responders and nonresponders to ICIs were observed in The Cancer Genome Atlas dataset. The prevalences were consistent with the exception of *A. calcoaceticus*, which was observed but not as prevalent as in the ORIEN dataset (Supplementary Fig. S4). However, the genus *Acinetobacter* was consistently prevalent between the two datasets. We observed no significant differences between α-diversity metrics of responders and nonresponders (Welch two-sample *t* tests *P* value >0.4; Fig. 3C). We found that the random forest classifiers based on microbe diversity measures with five rounds of fivefold cross-validation performed poorly relative to our other microbe-based classifiers (Fig. 3D).

### Correlation of tumor RNA-seq (GSEA) with microbes

**Figure 4:**
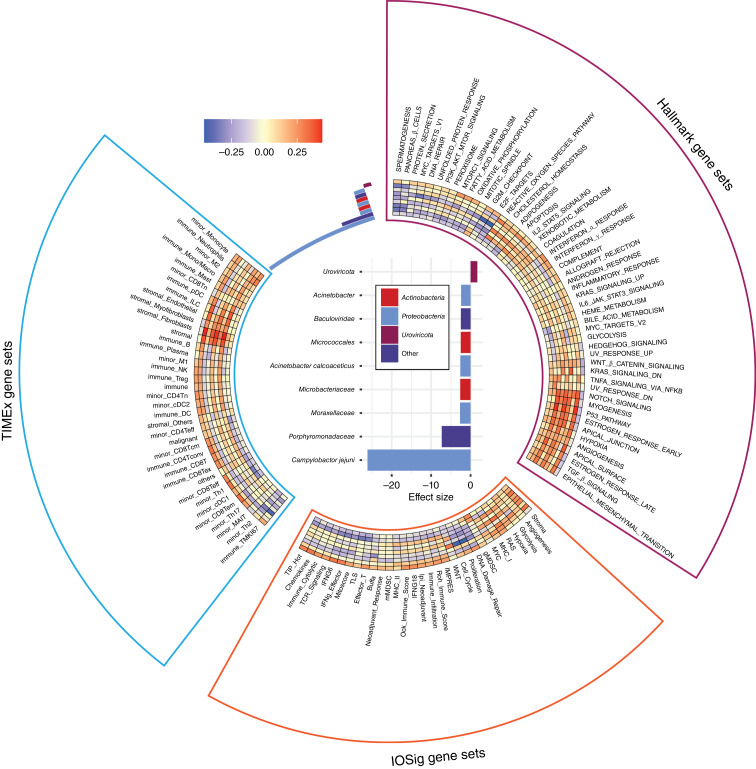
Association of microbes and gene signatures. Effect size plot showing the top nine most significantly enriched species. Spearman correlation coefficients between the significantly enriched species, the most significantly correlated signatures, and other gene sets shown in a heatmap.

We next asked whether microbe abundance in the tumor could be associated with tumor-intrinsic pathways or the composition of cell types in the TIME. We focused on the nine microbes identified to be differentially abundant in relation to immunotherapy response in melanoma. For the top nine microbes, one microbe associated with responders to immunotherapy and eight with nonresponders (Fig. 4). To investigate the intrinsic pathways that correlated with the microbes, we performed ssGSEA on patients with melanoma using MSigDB Hallmark gene sets. *Uroviricota*, which was found to be highly abundant in responders, was correlated with inflammation and immune-related gene sets and pathways (Fig. 4). Conversely, we observed that the microbes that were highly abundant in nonresponders, bacteria in the *Moraxellaceae* family, correlated most strongly with notch signaling–related, myogenesis-related, and p53 pathway–related genes (Fig. 4). The full list of adjusted *P* values is available in Supplementary Table S5. These results are consistent with our previous findings, in which we observed similar Hallmark gene sets and pathways enriched in responders versus nonresponders across five melanoma cohorts of immunotherapy-treated patients with pre- and on-treatment tumor biopsies (28).

To further dissect the association of microbe abundance and the composition of cell types in the context of immunotherapy responses in melanoma, we performed cell-type deconvolution using the bulk RNA-seq with TIMEx. We found that *Uroviricota* was highly correlated with the enrichment of tumor-infiltrated immune cell types, including CD8^+^ T cells, which are known predictors of immunotherapy response (Fig. 4). In contrast, the lack of tumor-infiltrated immune cell types was correlated with microbes associated with nonresponders. In particular, we observed that stromal cell types, such as fibroblasts and endothelial cells, were enriched in association with the microbes noted in nonresponders, the *Moraxellaceae* and *Microbacteriaceae* families (Fig. 4). The tumor immune cell composition corroborated our previous findings (28).

Next, we asked whether the microbe abundance was associated with any gene signatures predictive of immunotherapy responses. To investigate this question, we utilized 31 previously published gene signatures that have been indicated to be associated with immunotherapy responses (28). We correlated microbe abundance with these signatures and found that gene signatures associated with inflammation or immune activation were highly associated with microbes abundant in responders (Fig. 4). On the other hand, gene signatures associated with immune-suppressive or -intrinsic signaling were highly associated with microbes abundant in nonresponders (Fig. 4). These results suggest that microbe abundance could provide a different dimension in understanding the TIME in predicting immunotherapy responsiveness in melanoma.

### Prediction of response using tumor gene expression and microbe abundance

**Figure 5:**
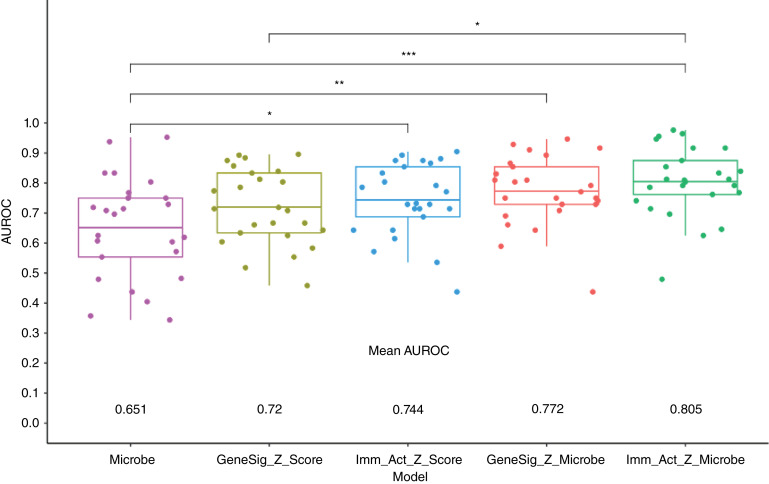
Prediction of response using gene expression and microbes. Mann–Whitney comparisons of the mean AUROC values from random forest model comparisons.

**Figure 6:**
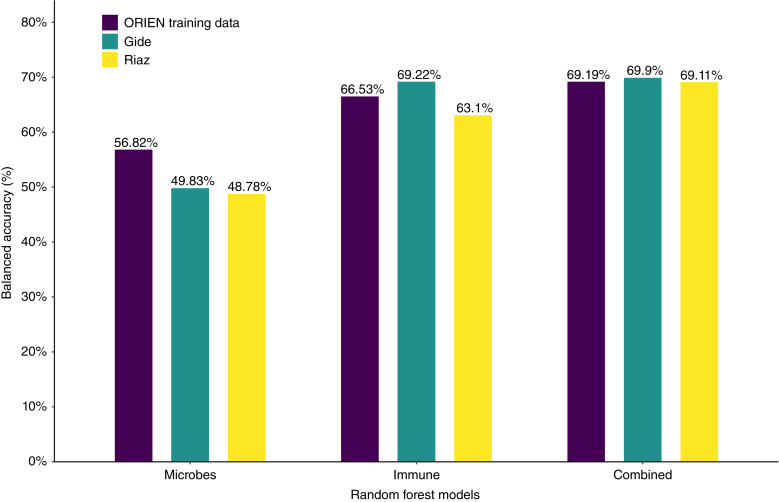
Prediction of random forest model combining microbe abundance and gene signatures. Balanced accuracies achieved by the microbe abundance, gene expression, and combined models for each of the three datasets.

We further hypothesized that combining microbe abundance features with gene expression signatures could improve response prediction of melanoma to immunotherapy. To test this hypothesis, we developed an ensemble learning random forest classifier using microbe abundance and gene signatures identified to be associated with immunotherapy responses in melanoma (see Supplementary Fig. S5 for testing of other machine learning approaches). We first developed the random forest classifier based on microbe abundance with 15 input features (microbe) and performed 5 rounds of fivefold cross-validation on the melanoma cohort (Fig. 5). The average AUROC for the microbe classifier was 0.651. We also constructed a random forest classifier based on 31 gene signatures (GeneSig_Z_score) or the 16 immune-activated gene signatures (Imm_Act_Z_score), and the AUROC values for GeneSig or Imm_Act classifier were 0.72 and 0.744, respectively (Fig. 5). Notably, when we combined the microbe abundance and gene signatures to develop the random forest classifier, the ensemble learning random forest classifier for gene signatures plus microbe (GS_Z_microbe) and immune-activated gene signatures plus microbe (Imm_Act_Z_microbe) achieved 0.772 and 0.805, respectively (Fig. 5). Finally, to validate the generalizability of this combined microbe and gene expression signature random forest classifier, we tested this signature in two independent previously published datasets of Gide and colleagues (32) and Riaz and colleagues (33). Raw RNA-seq data were downloaded from BioProject, processed, aligned, and quantified for the gene expression and microbe abundance according to Fig. 1. As illustrated in Fig. 6, we found that the combined microbe and gene signature random forest classifier achieved 69.9% and 69.11% balanced accuracy in Gide and Riaz datasets, respectively. This suggests that microbe abundance features provide a distinct layer of information in predicting response to immunotherapy and, when combined with gene expression signatures, can improve the prediction of response to immunotherapy in melanoma.

## Discussion

We utilized tumor RNA-seq from patients with melanoma to explore the tumor microbiome’s influence on clinical outcomes, specifically in response to ICIs. We observed microbes in all samples and showed that tumors that responded to ICIs had significantly different taxa present from those that did not respond to treatment. Consistent with previous findings, gene expression seems to be predictive of response to ICIs. In addition, we showed that microbes are also predictive of response to ICIs, particularly when combined with gene expression, suggesting that the inclusion of microbes in these models enhances predictive ability.

A correlation between the gut microbiome and response to ICIs has been consistently indicated in previous research (43–45). Altering the gut microbiome via responder-derived fecal microbiota transplantation has been shown to induce a clinical response to anti-PD1 treatment in patients with melanoma (46, 47). However, many of the efforts in this area have focused solely on the gut microbiome. Therefore, we assessed the tumor microbiome to further explore the impact of microbes on clinical outcomes in body sites beyond the gut.

We observed the presence of microbiota in all 71 tumor samples, as is consistent with previous findings with regard to the tumor microbiome (48, 49). Our study explicitly exhibits the microbial characteristics of tumors in patients with metastatic melanoma. Previous research has shown that the tumor microbiome in this specific subset of cancer is predictive of response to treatment, but these findings have been limited in scope because of samples having been collected before the use of modern ICIs as a standard treatment regimen for metastatic melanoma (20). We showed distinct, significantly enriched taxa, including fungi, at baseline for patients treated with contemporary ICI-based treatment plans.

The mechanisms by which tumor microbes affect response to ICIs may relate to interactions with the immune system or several other established mechanisms (50).The World Health Organization has officially recognized a causal association between 11 microbes and cancer (51). However, in recent years, the number of likely carcinogenic microbes and more loosely related “complicit” microbes has increased dramatically. These have been shown to interact with the host via diverse mechanisms. For example, in colon cancer, *Bacteroides fragilis* biofilms on colon polyps have been found to secrete a toxin that directly damages DNA (52, 53), as have some *Escherichia coli* (54). In another mechanism, *Helicobacter pylori* secrete a series of molecules eliciting an inflammatory cascade shown to drive tumorigenesis in gastric adenocarcinoma and mucosa-associated lymphoma (55, 56). The fungal genus *Malassezia* caused pancreatic ductal adenocarcinoma growth through activation of the C3 complement pathway (57). The microbe enriched in responders has a precedence for interacting with the human immune system in the context of cancer. *Uroviricota,* which contains diverse bacteriophages, has been shown to contain antigens with cross-reactivity to melanoma tumors (58). On the other hand, *C. jejuni* has not been associated with the tumor microbiome or response to ICIs, although it is an established pathogen that has been linked to food-borne illness (59). In our study, it is associated with the distinct immune expression pathways as *Uroviricota*, suggesting that it acts through a very different mechanism. Furthermore, recent studies have identified bacteria-derived HLA-bound peptides in melanoma presented by tumor cells could elicit immune reactivity. This intratumoral bacteria peptide repertoire could be further explored to understand the mechanism by which microbes modulate the immune system and responses to therapy (60). The demonstration of the utility of high-throughput sequencing to explore these correlations warrants a broader search.

Efforts have been made to identify predictors of response and resistance to ICIs. As previously discussed, expression signatures have been established as predictors of ICI response in metastatic melanoma (9, 12, 14, 15, 28, 61). One such study assessing the model combining IFNγ and TMB found that it was predictive of response but not resistance (61). Another such study developed a multiomics-based classifier that successfully predicted response but was unable to predict resistance (20). We showed significantly enriched taxa in both response groups. We also showed that microbes alone are predictive of response/resistance to immunotherapy and when combined with gene expression, enhance the model’s predictive ability. These accuracies are consistent with other studies. For example, Mihály and colleagues reported that the best performing genes for predicting OS had AUC values of 0.64, 0.62, and 0.62 (62). Liu and colleagues reported that the AUC values for “1-year, 3-year, and 5-year OS were 0.832, 0.850, and 0.768; 0.712, 0.591, and 0.602; and 0.773, 0.702, and 0.673 for the training set, testing set, and whole set, respectively,” for their 6-gene signature (15). Long and colleagues reported that the AUC values of their 4‐gene biomarker prognostic model were 0.7561, 0.7674, 0.7366, 0.7040, and 0.6919 (63). Although these values are too low for clinical relevance, they provide a proof of concept that the inclusion of tumor microbes in predictive models can improve accuracy. Although a more rigorous approach to these predictive models would include a subgroup analysis on the ICI treatment types, we still see predictive power on the broader groups, providing evidence that we could harness yet more predictive power with the inclusion of treatment-type information. Further studies are warranted to combine tumor microbiome abundance with other clinical and “omics” (e.g., genomics and pathomics) for developing an accurate classifier for predicting immunotherapy responses in melanoma. Our findings also warrant further research to evaluate whether these correlations are causally associated with outcomes and their effect on the TIME and immune cell infiltration.

Although there is much utility in assessing a real-world clinical dataset, it also presents significant limitations. Notably, critical information such as stage, progression-free survival, Eastern Cooperative Oncology Group score, and prior treatment were unavailable. Additionally, although the majority of patients were treated with anti-PD1/PDL1 inhibitors, a small subset differed in treatment regimen. This introduces a major limitation as a stratified analysis could provide a meaningful insight, but the study is not sufficiently powered for this because of its small sample size. Previous studies have observed different microbes affecting anti-CTLA4 versus anti-PD1/PDL1 responses owing to distinct mechanisms of action. For instance, *Bacteroides* may induce an antitumor immune response in anti–CTLA4-treated tumors by activating dendritic cells in the gut to produce IL12, thereby stimulating T helper cells (64). It has been suggested that in anti–PDL1-treated tumors, microbes found in the gut may modulate the immune response through mechanisms such as secretion of small molecules such as inosine or indole-3-acetic acid (65, 66). However, tumors with evidence of immune activation (as measured by T-cell infiltration, PDL1 expression, IFNγ-related gene expression, or high TMB) have been shown to be susceptible to ICIs whether anti-PD1 treatment was used as monotherapy or in combination with anti-CTLA4 antibodies (1, 67). Therefore, intratumoral microbiome features that enhance the immunogenicity of the TME are expected to make these tumors more susceptible to the ICI regimens included in our cohort. Furthermore, we have no means by which microbes are assessed as intra- or extracellular. We note that both have been observed by microscopy (19), though the intra–cancer cell observations have recently been called into question (not, however, the intra–immune cell observations; ref. 68). Further studies are needed to confirm the location of tumoral microbes. Our findings also raise the continued question of whether additional filtering steps to control for contamination are required. Recent work suggests that more stringent human filtering, including the removal or masking of contaminating human reads in draft microbial genomes, is necessary. Many of the associations are conducted on a large number of microbes and genes or signatures, leading to many independent tests. Multiple hypothesis correction is likely insufficient to remove all false discoveries, driving further need for experimental validation. Finally, we validated the combined microbe and gene expression random forest classifier in two independent datasets and showed that the balanced accuracy is about 69%. We acknowledge that there is room for improvement for the machine learning classifier, especially testing the model, on more independent datasets to confirm the value of adding microbes when overfitting can be more accurately assessed.

In conclusion, we found that the tumor microbiome in patients with metastatic melanoma was significantly different in those that responded (>24 months survival) to treatment with ICIs from those who did not respond. Furthermore, the microbial communities had the ability to predict response when incorporated into machine learning models. The tumor microbiome further enhanced models to predict response when combined with gene expression data. Future research has the potential to support therapeutic strategies to modify the tumor microbiome to improve ICI treatment outcomes.

## Supplementary Material

Figure S1 Association of CIBERSORT cell types with the response to ICIs

Figure S2 16 gene signatures where high Z-scores are associated with ICI responsiveness in this cohort (FDR < 0.05)

Figure S3 Predictive values (AUROC) of the 16 gene signatures in the ORIEN cohort (IO_NOVA_Mel) and 22 other melanoma cohorts

Figure S4 Comparison with TCGA for microbes most significantly enriched in response groups

Figure S5 Comparison of alternate machine learning methods

Table S1 The list of published gene signatures compared between treatment responders and non-responders and to tumor microbe abundances.

Table S2 Modeling results for gene expression differences between responders and non-responders

Table S3 Model performance of 31 published gene expression signatures for predicting immunotherapy treatment response

Table S4 Modeling results for the microbes enriched in the tumors responsive or not-responsive to immunotherapy

Table S5 Correlations between microbe abundances and MSigDB Hallmark gene sets average Z scores
