# Supplementary figures and images for: The Tumor Microbiome as a Predictor of Outcomes in Patients with Metastatic Melanoma Treated with Immune Checkpoint Inhibitors
Source: Cancer Res Commun. 2024 Aug 8;4(8):1978–90. doi: 10.1158/2767-9764.CRC-23-0170 (PMC11307144; doi:10.1158/2767-9764.CRC-23-0170)

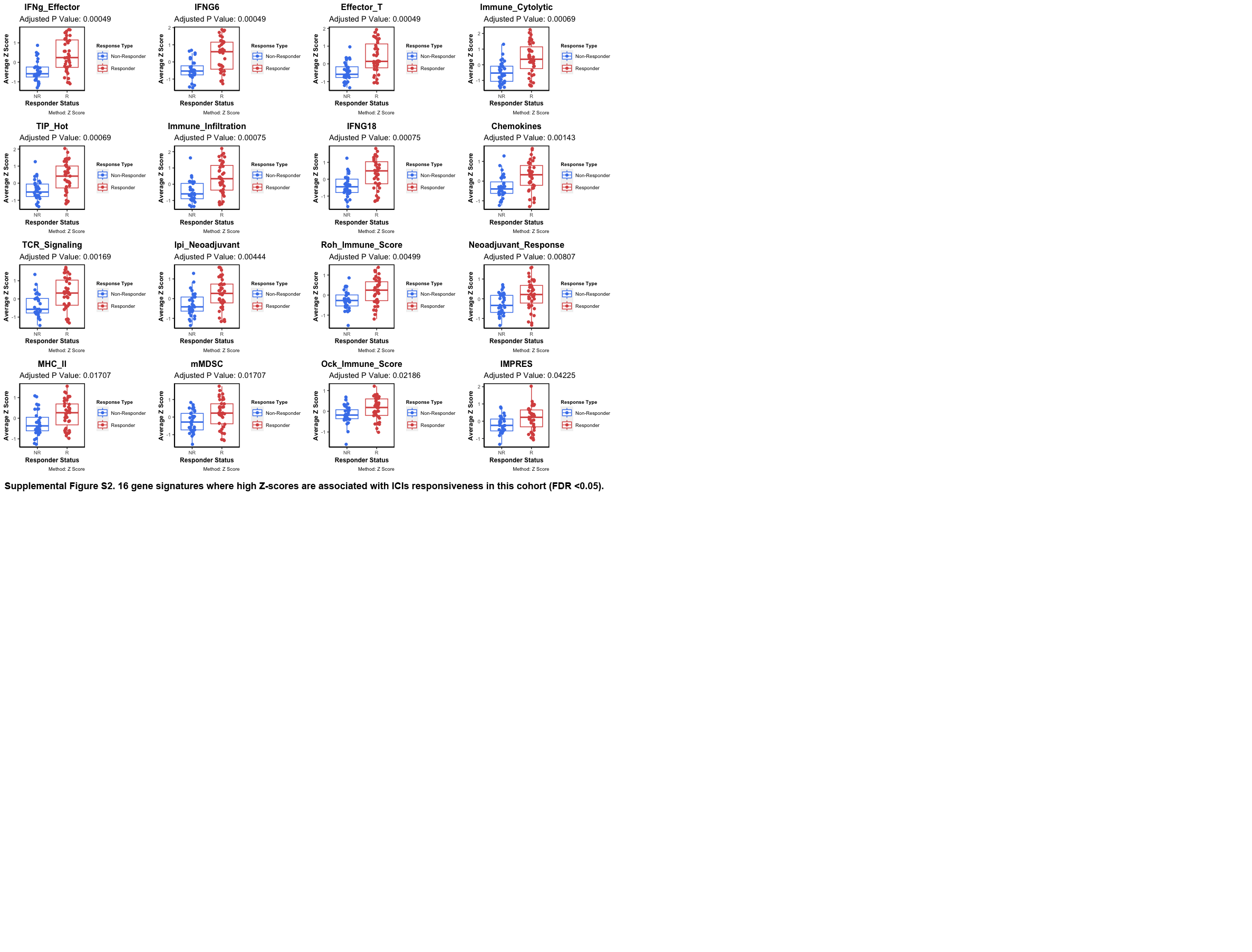

Supplement: Figure S2 — 16 gene signatures where high Z-scores are associated with ICI responsiveness in this cohort (FDR < 0.05) [file crc-23-0170_figure_s2_supps2.png]

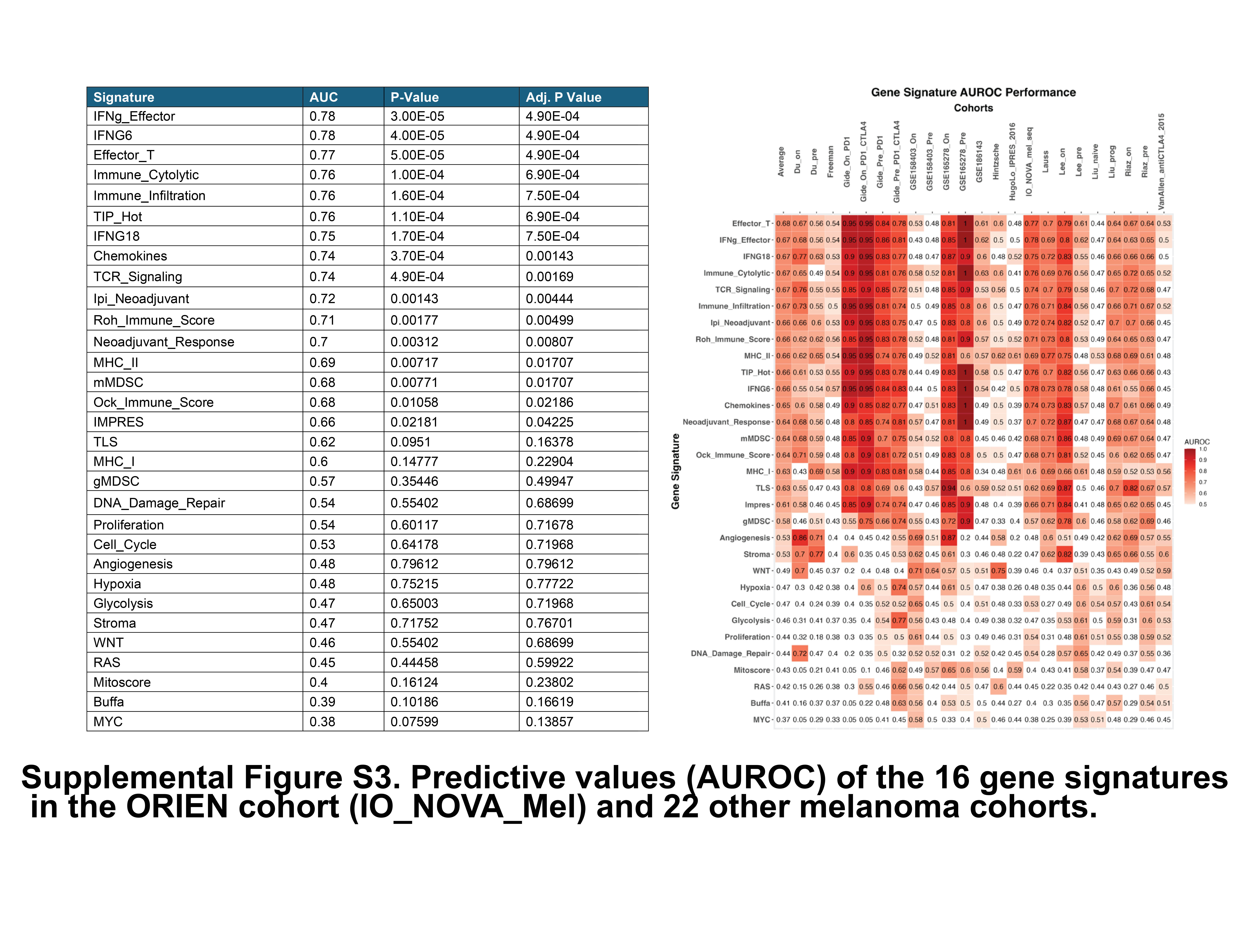

Supplement: Figure S3 — Predictive values (AUROC) of the 16 gene signatures in the ORIEN cohort (IO_NOVA_Mel) and 22 other melanoma cohorts [file crc-23-0170_figure_s3_supps3.png]

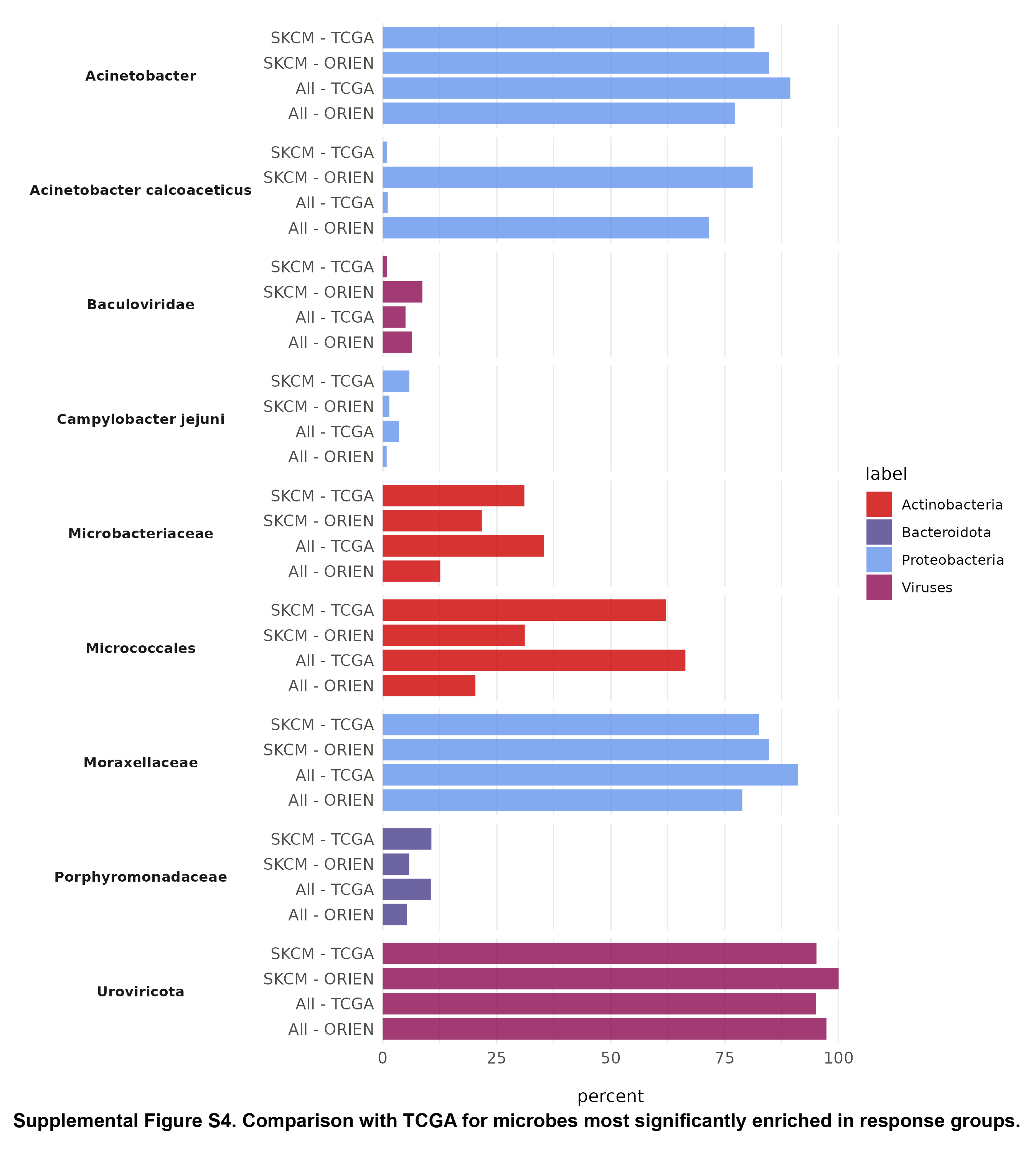

Supplement: Figure S4 — Comparison with TCGA for microbes most significantly enriched in response groups [file crc-23-0170_figure_s4_supps4.png]

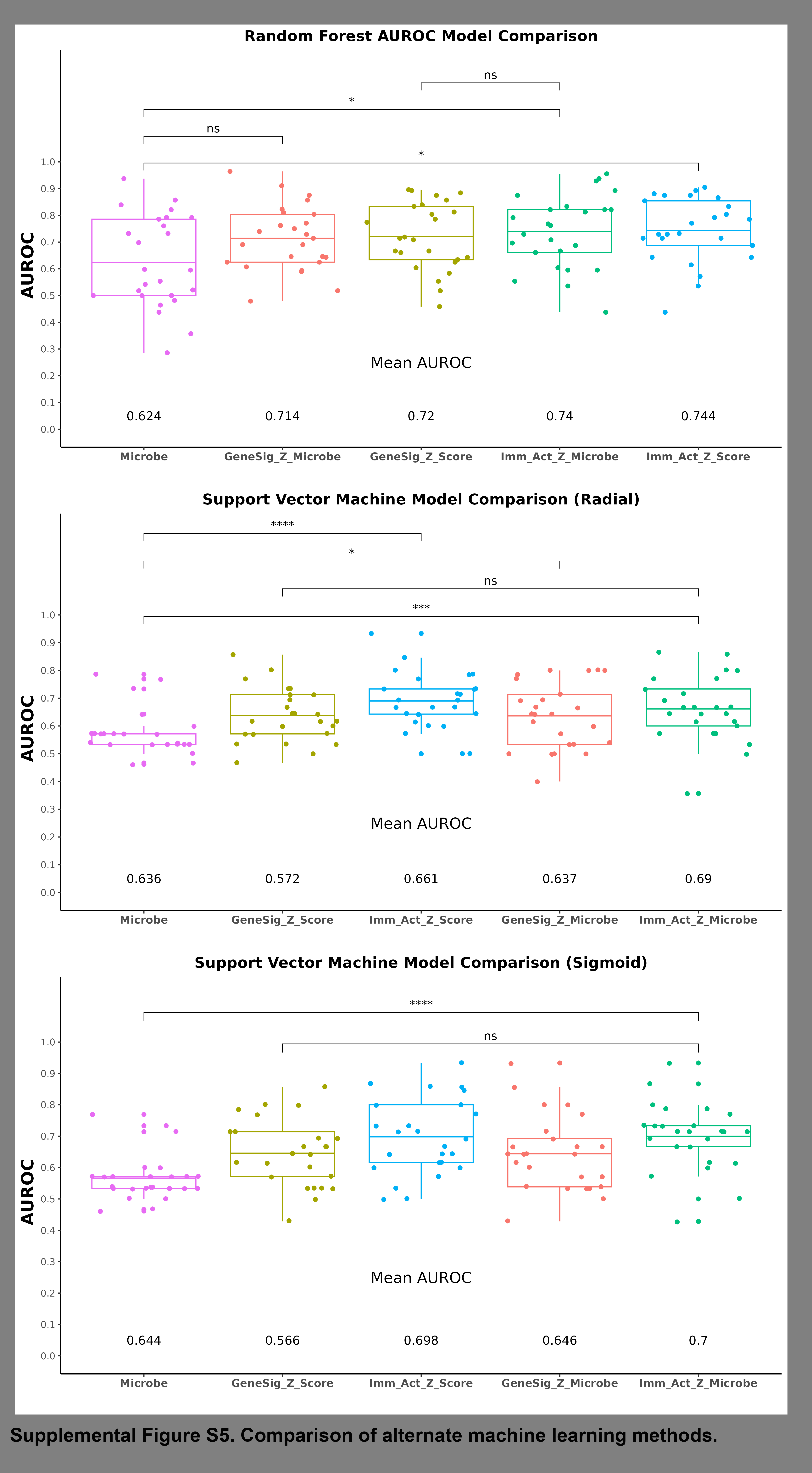

Supplement: Figure S5 — Comparison of alternate machine learning methods [file crc-23-0170_figure_s5_supps5.png]
